# Understanding social integration and health outcomes among China's internal migrants: a systematic review

**DOI:** 10.3389/fpubh.2025.1536526

**Published:** 2025-03-05

**Authors:** Xiao Yang, Yuxuan Li, Kun Tang

**Affiliations:** ^1^Department of Health Policy and Management, Johns Hopkins Bloomberg School of Public Health, Baltimore, MD, United States; ^2^Vanke School of Public Health, Tsinghua University, Beijing, China; ^3^School of Basic Medical Sciences, Tsinghua University, Beijing, China

**Keywords:** migrant population, China, health service utilization, self-reported health, noncommunicable disease, social integration

## Abstract

**Background:**

The internal migrant populations in China have contributed significantly to the country's social and economic landscape, yet health disparities among migrants remain a pressing concern. Social integration is considered a critical factor influencing health outcomes, but evidence on this relationship is fragmented. This systematic review aims to synthesize existing studies to explore the association between social integration and health outcomes, including healthcare service utilization, self-reported health (SRH), and non-communicable disease (NCD), among internal migrants in China.

**Methods:**

A systematic search was conducted across three databases (PubMed, EMBASE, PsycINFO) to identify studies published from January 2014 to November 2024 according to the protocol (INPLASY2024110085). The JBI assessment tool was used to assess the quality of the included studies.

**Results:**

A total of 16 eligible cross-sectional studies were identified, focusing on the multidimensional aspects of social integration, including economic integration, acculturation, social networks and self-identity and their influence on health outcomes. Eleven papers focused on health service utilization, four concentrated on SRH and one discussed both NCDs and SRH. The majority of included studies indicated a significantly positive association between social integration and health outcomes of internal migrants.

**Conclusion:**

Social integration plays a crucial role in addressing health disparities among internal migrants in China. This review emphasizes the need for initiatives at all levels to enhance economic inclusion, cultural adaptation, and social networks to foster better social integration for the migrant community. Addressing these gaps will promote equitable healthcare access and improve the wellbeing of internal migrants in China.

**Systematic review registration:**

Identifier INPLASY2024110085, https://inplasy.com/inplasy-2024-11-0085/.

## 1 Introduction

Improving the health of migrants is a global priority, aligning with international frameworks such as the One Health Approach and the United Nations 2030 Agenda for Sustainable Development (1, 2). Migration, driven by urbanization and economic shifts, presents significant public health challenges, necessitating policies that ensure equitable healthcare access and social protection. The One Health Approach, which recognizes the interconnectedness of human, animal, and environmental health, illustrates the importance of addressing migrant health as part of a broader public health strategy (1). Similarly, the UN Agenda 2030 emphasizes health equity and social inclusion through Sustainable Development Goal (SDG) 3 (Good Health and Wellbeing) and SDG 10 (Reduced Inequalities), emphasizing the need to integrate migrants into healthcare and social systems (2). Ensuring that migrants receive adequate healthcare and social support is not only critical for their wellbeing but also essential for sustainable and inclusive development at national and global levels.

As one of the most populous developing countries in the world, China has a significant internal migrant population (3, 4). The existence of internal migrants is crucial to China's poverty reduction and economic development, and they serve as the primary labor force role in the continuous urbanization process of the country (5). Although in the past few decades, migrants have basically been composed of low-educated, heavy manual laborers, recent years have seen an increase in highly skilled, educated technical professional white-collar workers among the migrant population (6, 7). This emerging trend also indicates higher expectations for the social welfare and health status of the migrants in the destination region.

However, China's household registration system (“*Hukou*”) limits the migrants from obtaining permanent citizenship in the place of inflow, leading to socioeconomic inequalities in multiple dimensions including income, housing, social welfare, education, and healthcare services between the migrants and the native residents (8–12). To address the gaps, the Chinese government has implemented a variety of policy measures in recent years (13, 14). In terms of health care, the New Rural Cooperative Medical Insurance System (NRCMS) aiming to reduce disparities in healthcare access has been developed by the Chinese government for those who have rural *Hukou* and has successfully covered a majority of the rural-to-urban populations (15–17). However, despite these efforts, migrant populations still show relatively low utilization rates of healthcare services and low health awareness compared to urban residents (18, 19).

Research on the health of China's migrant population has focused primarily on infectious diseases, mental health, and occupational health (20). Studies show a high incidence of infectious diseases among the migrant population, compounded by limited preventative measures (21). Specifically, the immunization rates of the internal migrants are lower than that of the local residents, raising the risk of outbreaks, and they are at higher risk for communicable diseases, such as HIV and tuberculosis, with poorer cure rates and significant more drug-resistant tuberculosis incidence compared to the general population (22–27). The migrant populations are also prone to adverse mental health conditions due to low socioeconomic status, high work pressure, and separation from home. This results in a higher likelihood of depression, reduced wellbeing, and substance abuse, even correlating with an increased suicide risk (28–31). Occupational hazards including injuries, musculoskeletal disorders, and chronic poisoning are major ailments among internal migrants that stem from heavy physical labor and lack of protective awareness (32–34). As China's migrant population ages, the NCD burden grows as well (35). While some studies show no significant difference in NCD prevalence between migrants and the natives, others indicate a higher prevalence among the migrants, though with lower treatment and awareness (36–38). Given poorer lifestyle habits and limited NCD management knowledge, this group may face increased chronic disease morbidity in the long-term (39, 40). Considering that migrants constitute a health-vulnerable population, it is essential to systematically examine the determinants influencing their health outcomes.

Social integration refers to the process through which migrant individuals or groups gradually adapt to, accept and form a stable relationship with a new society, and encompasses various aspects including identity, social acceptance, acculturation and socioeconomic status integration (41, 42). Social integration focuses on the inclusion process of individuals or groups of expats into a new society, which comprises inclusion at various levels, from the societal environment and policy acceptance at the broader spectrum, community support and cohesion, to direct interpersonal relationships in day-to-day lives (43, 44). Social integration is also a journey that involves different phases, from the initial marginalization to preliminary adaptation and gradual integration, and ultimately reach full assimilation to the host society (45). Certainly, some migrants may choose different integration strategies and may halt this process at a particular stage (45).

In international migrant research, another term often used interchangeably with social integration is acculturation (46). While both processes require individuals to adapt to new environments, social norms, and contribute to a sense of belonging, acculturation centers more on the culture adaptation process of the migrants in their place of settlement, especially in language and customs (47). While both social integration and acculturation reflect proactive behaviors initiated by migrants to adapt to the host environment, social support, on the other hand, is a more involuntary experience perceived by migrants as an indication of the host society's acceptance of them, which is also an indispensable element of the integration process, providing essential emotional and material resources that facilitate the adaptation and contribute to a sense of belonging (48).

The role of social integration in migrant health is complex. Evidence from international research suggests that the social integration status of immigrants in their new settlements is significantly associated with their physical and mental wellbeing, which gives rise to the “healthy migrant effect” hypothesis. This hypothesis proposes that migrants initially exhibit health advantages but may experience a gradual decline in health over time as they integrate into local communities (49–51). This decline may be related to the stress, social adaptation challenges, lifestyle changes and downward shifts in socioeconomic status faced by immigrant groups in their settlements (52–55). Studies have shown that the integration process with the local society will bring pressure to immigrants and affect their mental health and even physical markers such as blood pressure levels (56–59). Furthermore, the degree of social acceptance influences migrants' employment opportunities, and the intensity of labor and income can determine their occupational health and lifestyle (60–62). In addition, the availability of health education resources and healthcare services for newcomers in their place of residence may also impact their risks of infectious and chronic diseases, as well as maternal and child health outcomes (63–68). Therefore, social integration may be a critical factor in migrant health.

Social integration extends beyond inclusion to encompass social equity, both of which are fundamental to the wellbeing of migrants. The United Nations advocates that the goal of social integration should aim to “create a more stable, safe, and just society for all” (69). Achieving this requires not only ensuring that migrants are included but also guaranteeing that they receive fair treatment in policies, healthcare access, employment opportunities, and social welfare, comparable to that of local residents. However, both internal migrants in China and migrants who move internationally continue to face systematic barriers to health stemming from social justice issues, which in turn further exacerbate existing social inequalities (70–73). This persistent disparity contradicts the significant contributions migrants make to the host society, where they play a vital role in economic and social development (74). Recognizing migrants as integral members rather than outsiders is essential to developing a more inclusive and equitable society. Therefore, ensuring their equal rights to healthcare and wellbeing is not only a matter of justice but also a crucial factor in promoting social cohesion, sustainable development, and long-term stability in host communities (75).

Despite the current literature on China's migrant population health and international evidence linking social integration with health outcomes, to our best knowledge, there has been no systematic review that explored the available literature on the association between social integration status and the health outcomes among internal migrant populations in China. Therefore, this study aims to identify and summarize existing research on social integration and its relevant health outcomes of China's migrant population through a systematic review, with a focus on the healthcare service utilization, self-reported health (SRH) and noncommunicable diseases (NCDs), to provide policymakers more comprehensive evidence of the actual needs of migrant populations in terms of social integration and health, support the development of relevant policies that reduce health inequalities, enhance the health and wellbeing, and promote social integration of migrant communities.

## 2 Methods

### 2.1 Study protocol registration and search strategy

For the transparency of the study, the protocol of this systematic review has been registered with the International Platform of Registered Systematic Review and Meta-analysis Protocols (INPLASY; INPLASY2024110085). The systematic review is conducted followed the guidance of the “Preferred Reporting Items for Systematic and Meta-analysis Statement 2020” (*PRISMA 2020*) (76). The search for the literature was conducted on 11th November 2024. To identify relevant literature, three international databases (PubMed, EMBASE, PsycINFO) were included for the search using different combinations of the search terms listed in Table 1. When conducting the search, the main search terms are combined using the “AND” function, while the alternative expressions within each main term were searched using the “OR” function. For the alternative expressions of “Chronic diseases,” “Cardiovascular disease,” “Chronic respiratory disease,” “Cancer” and “Diabetes” were listed as they are the four most prevalent NCDs in China (77).

**Table 1:** List of search terms and synonyms.

**Main terms**	**Synonyms/alternative expressions**
Migrant population	Migrants
	Floating population
	Migrant workers
	Internal migrants
	Nongmingong
	Rural-to-urban migrants
Social integration	Assimilation
	Social adaptation
	Social network
	Social cohesion
	Social support
	Social engagement
	Acculturation
	Social Inclusion
Chronic disease	Non-communicable disease
	Cardiovascular disease
	Chronic respiratory disease
	Cancer
	Diabetes
Self-rated health	Self-reported health
	SRH
Healthcare	Health service
China	Chinese

### 2.2 Inclusion and exclusion criteria

Quantitative studies published in English focusing on the internal migrant population in Mainland China of both sexes were considered for inclusion. Since the past 10 years have been a prosperous period for migrant health research in China, studies published between January 2014 and October 2024 that met the inclusion criteria were included. To further ensure the accuracy of the review, we have established a series of detailed exclusion criteria based on the PICOS (Participant, Intervention, Comparison, Outcome and Study Design) principle. Studies on migrant children participants who are under 18 years old were excluded from the review, as their social networks are mainly regulated by their parents. Studies in other languages, have a focus on the Chinese immigrants abroad or international immigrants to China were excluded. Qualitative studies, narrative reviews, systematic reviews and/or meta-analyses, case reports, and studies with only abstracts or protocols available were also excluded.

### 2.3 Study screening and data extraction

The database search was conducted independently by two reviewers (X.Y. and Y.L.). After collecting the overall records of all databases, literature deduplication was first performed before literature screening. Guided by the inclusion and exclusion criteria, one reviewer (X.Y.) independently examined the titles and abstracts to determine eligibility for full-text screen. Then, two reviewers assessed the full article for potential inclusion independently. Discussions were carried out if there were discrepancies between the two reviewers and the decision was made until a final consensus was reached. Ultimately, the reviewers reviewed the reference lists of the studies included and employed a snowballing method to identify and incorporate additional relevant studies from those references. Once a specific study was selected, the information was extracted by one reviewer (X.Y.) as follows: author information, publication year of the research, type of research, study population, sample size, type of health outcome and the strength of the association. All data extraction was conducted using standardized data extraction table pre-developed by the research team.

### 2.4 Appraisal of study quality

The quality of each selected article was assessed using the *JBI Critical Appraisal Tool for Analytical Cross-Sectional Studies*, which checklist evaluates each cross-sectional study with 8 items including inclusion criteria, description of study subjects and settings, description of exposure, measurement of the condition, identification and control of confounding factors, the reliability of the outcome measures and the appropriateness of statistical analysis (78). Based on these dimensions, two reviewers independently evaluated the studies based on the 8 criteria. Each criterion was assigned a score of 2 (fully met the criteria), 1 (partially met the criteria), or 0 (did not met the criteria or not applicable), respectively, and the summed total score was between 0 and 16. Ultimately, each study was categorized as high quality (13–16 points), moderate quality (9–12 points) or low quality (0–8 points).

### 2.5 Data synthesis and visualization

The results of this systematic review were presented through a combination of narrative synthesis and graphical representations, enabling a comprehensive understanding of the research findings. The narrative synthesis offered an overarching perspective on the general characteristics of all included studies, while the graphical representations provided detailed insights into the specific aspects of each study. This narrative synthesis focused on elucidating the effects of social integration across three major dimensions: healthcare service utilization, SRH, and NCDs. Specifically, within the dimension of healthcare service utilization, we examined five key aspects: health record establishment, health education, preventive services, community health center utilization, and medical return behavior. Furthermore, the meta-analysis method was not considered suitable for this systematic review due to the limited number of included studies and the heterogeneity of research basic characteristics. Consequently, the narrative synthesis was deemed sufficient to effectively map the fundamental characteristics and emerging trends of the existing literature in this field of research.

## 3 Results

### 3.1 Study retrieval and screening results

**Figure 1:**
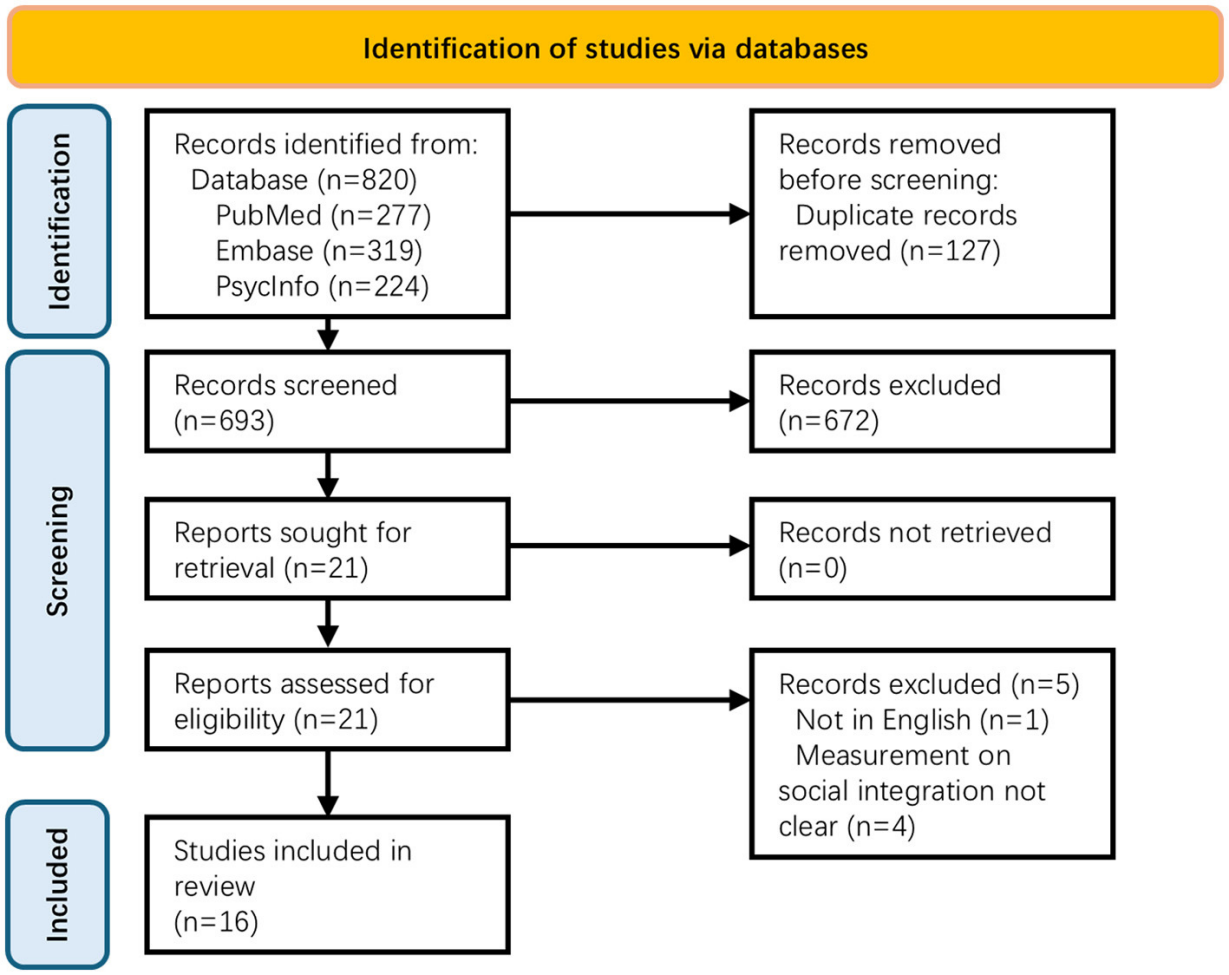
PRISMA 2020 flow diagram.

According to the search strategy, the database search yielded 820 publications (PubMed = 277; EMBASE = 319; PsycINFO = 224). After removal of duplicated records, a total of 693 publications remained for title and abstract screening and 21 publications were identified for full-text screening. Five papers were excluded and a total number of 16 publications were included for final review. Figure 1 shows the *PRISMA2020* diagram of the study selection process.

### 3.2 Characteristics of included studies

The 16 included studies were published between 2016 and 2023, and all studies employed a cross-sectional design. The sample size varied significantly from 307 to 169,989. While a majority number of studies explored the health outcomes on the general migrant population (*n* = 9), others had a focus on the most vulnerable migrant groups, such as the Older adult migrants (*n* = 4), female migrants (*n* = 2) and those who have been hospitalized (*n* = 1). Most of the studies used data from nationally representative migrant survey databases (*n* = 11). While 14 studies investigated within the migrant populations, two studies compared the migrant population and their local peers. Eleven studies centered on health service utilization; four studies dedicated on SRH while one study discussed both NCDs and SRH. The basic characteristics of the 16 included studies are detailed in Table 2, which presented the author's name and year of publication, study population characteristics, study design, measures of social integration, the health outcomes measured, covariates and study results.

**Table 2:** Basic characteristics of studies included in system review (*N* = 16).

**References**	**Study population characteristic (source database)**	**Study design**	**Measures of social integration**	**Outcome**	**Covariates**	**Study results**
**Health service utilization**						
Hou et al. (79)	15,999 migrants from 8 cities (2014 NIMPDMS)	Cross-sectional study	Structural social capital, including participation in organizations and participation in social activities. And neighborhood characteristics, including community urban status and neighborhood composition	Health record establishment; Health education receipt	Gender, age, marital status, educational attainment, monthly income, employment status, Hukou status, migration type, years lived in the destination regions, self-reported health	Migrants engaged in social organizations (OR = 1.467, 95% CI: 1.201–1.793) and social activities (OR = 1.620, 95% CI: 1.329–1.976), as well as those with more local neighbors (OR = 1.329, 95% CI: 1.050–1.682) or residing in urban communities (OR = 1.735, 95% CI: 1.224–2.460), were more likely to establish health records.
Hu et al. (80)	307 married migrant women in Changsha city, Hunan Province	Cross-sectional study	Social Support Rate Scale (SSRS), including subjective social support, objective social support, and utilization of social support	Health record establishment; health education receipt; free contraceptive service; Cancer screening	Family location, migration time in Changsha, migration time in total, living circumstances and years of education	Married migrant women with greater objective social support were more likely to receive health education (OR = 1.15; 95% CI: 1.04–1.26). Higher subjective support was associated with contraceptive services (OR = 1.11; 95% CI: 1.05–1.18) and cancer screening (OR = 1.10; 95% CI: 1.02–1.17). Greater social support utilization was linked to health record establishment (OR = 1.24; 95% CI: 1.06–1.44), contraceptive use (OR = 1.21; 95% CI: 1.04–1.42), and cancer screening (OR = 1.29; 95% CI: 1.10–1.51).
Jiang (81)	148,338 migrants in China (2017 CMDS)	Cross-sectional study	Social cohesion: Objective cohesion supervising community work, making proposals to local governments, participating in donations or volunteer work, and joining political party events Subjective cohesion: attitude of the inflow place, attention to local changes, attitude of integrating into local life, and willingness to be local members	Health record establishment; health education receipt	Sex, age, Hukou, community type, self-rated health, migration range, migration time, migration reason	Both objective and subjective cohesion forms were positively associated with basic public health services utilization, specifically, for each unit of the increase of the following item, the probability of the healthcare utilization increased: Objective Cohesion: Supervising community work: +24.1% health records, +0.129 health education (*p* < 0.001) Propose to government: +0.062 health education (*p* < 0.001) Donations/volunteering: +27.5% health records (*p <* 0.001), +0.192 health education (*p <* 0.001) Political party activities: +5.8% health records, +0.026 health education (*p <* 0.05) Subjective Cohesion: Liking the inflow place: +6.4% health records (*p <* 0.05) Attention to local changes: +20.3% health records, +0.135 health education (*p <* 0.001) Willingness to be a local member: +10.6% health records, +0.082 health education (*p <* 0.001)
Jing et al. (82)	3,138 older adult migrants > 60 y.o. (2015 NIMDMS)	Cross-sectional study	Social integration is measured through three dimensions: economic integration, including average monthly household income, occupation and local medical insurance; social interaction, including number of local friends, and self-identity, including settlement willingness	Health record establishment	Hukou, age, time in inflow area, movement area and exercise time per day	Older adult migrants with local medical insurance (*p < * 0.001, OR = 2.03), plans to settle in their current residence within the next 5 years (*p <* 0.001, OR = 1.37), and more than three local friends (*p <* 0.001, OR = 1.54) were more likely to establish health records
Liang et al. (83)	154,008 migrants (2017 CMDS)	Cross-sectional study	Social integration is measured by economic integration (employment and residential pattern), structural integration (social organizational participation, civil activities engagement, local medical insurance), sociocultural adaptation (local friends existence), and self-identity dimensions (settlement willingness)	Health record establishment; health education on the prevention of IDs and NCDs	Age, gender, marital status, education, family monthly income, Hukou, length of stay and the range of migration	More likely to establish health records: having a local house (OR = 1.082, 95% CI: 1.047–1.118), participation in social organizations (OR = 1.618, 95% CI: 1.576–1.660), civil engagement (OR = 1.355, 95% CI: 1.320–1.390), local medical insurance (OR = 1.321, 95% CI: 1.283–1.361), local friends (OR = 1.118, 95% CI: 1.085–1.151), and willingness to settle (OR = 1.247, 95% CI: 1.204–1.291); More likely to receive ID health education: Employed (OR = 1.121 95% CI: 1.081–1.163), participation in social organizations (OR = 1.700, 95% CI: 1.658–1.744), civil engagement (OR = 1.535, 95% CI: 1.497–1.574), local medical insurance (OR = 1.064, 95% CI: 1.033–1.095), local friends (OR = 1.146, 95% CI: 1.114–1.179), and willingness to settle (OR = 1.180, 95% CI: 1.141–1.220); More likely to receive NCD health education: having a local house (OR = 1.048, 95% CI: 1.016–1.081), participation in social organizations (OR = 1.807, 95% CI: 1.763–1.851), civil engagement (OR = 1.605, 95% CI: 1.566–1.644), local medical insurance (OR = 1.049, 95% CI: 1.020–1.079), local friends (OR = 1.150, 95% CI: 1.119–1.182), and willingness to settle (OR = 1.172, 95% CI: 1.135–1.211)
Lin et al. (84)	1,544 older adult migrants ≥60 y.o. from 8 cities (2015 NIMDMS)	Cross-sectional study	Social contacts: number of local friends and exercise time everyday	Free medical examination at community health center within the past year	Age, gender, education, marital status, medical insurance, average monthly household income, self-perceived health	Respondents with local friends had 2.859–4.607 times higher odds (*p* < 0.001) of using primary health care than those without local friends. Those exercising 61–90 min daily were more likely to utilize primary health care (OR = 3.515, 95% CI: 1.538–8.032) compared to those exercising less.
Wang et al. (85)	13,998 migrants (2017 CMDS)	Cross-sectional study	Subjective social integration status, including liking current location, noticing local changes and willingness to integrate; Subjective social exclusion status, including feeling accepted by locals, and senses locals look down on outsiders	Influenza vaccination rate	Education, health security card, income status, general health status; hypertension; Type 2 diabetes mellitus; the possession of an established health record; and having knowledge of the basic public health services program	Significant relationship between social integration and influenza vaccination rate (OR = 1.142; 95% CI: 1.04–1.22); no significance between social exclusion and influenza vaccination rate (OR = 1.062; 95%: 1.00–1.13)
Wang et al. (86)	169,989 migrants (2017 CMDS)	Cross-sectional study	Social communication, including social organization participation, civil activities engagement and local medical insurance; Acculturation: including preference of residence, the influence of hometown customs on migrants, and differences of health habits between migrants and local people Self-identity, including integration willingness and evaluation of identity	Utilization of primary health care among migrants: the receiving of health education on IDs and NCDs, and the first visit institution when migrants were sick	Sex, age, regions, marital status, educational attainment, and household registration status, length of migration, reasons of migration, and range of migration	Social Organization Participation: Migrants involved in social organizations were more likely to receive ID&NCD health education (OR = 2.5; 95% CI, 2.50–2.61; OR = 2.02; 95% CI: 1.98–2.06, respectively). Those who did not participate were less likely to choose Community Health Centers (CHCs) for primary care (OR = 0.94; 95% CI: 0.91–0.98). Civil Activity Engagement: Migrants engaged in civil activities the most often had lower odds of receiving ID health education (OR = 0.47, 95% CI: 0.35–0.63) and were more likely to use CHCs for primary care (OR = 2.61; 95% CI: 1.49–4.58). Local Medical Insurance: Migrants with local medical insurance were less likely to receive ID health education (OR = 0.76; 95% CI: 0.70–0.82; OR = 0.92; 95% CI: 0.89–0.96, respectively). Local identification: Migrants with strong local identification were less likely to use CHCs (OR = 0.66; 95% CI: 0.62–0.70). Preference of Residence: Migrants who like their inflow place had lower odds of receiving ID education (OR = 0.95; 95% CI: 0.92–0.97) and tended not to go to CHCs (OR = 0.96; 95% CI: 0.91–1.00). Hometown Customs Influence: Migrants least influenced by hometown customs were more likely to receive both ID (OR = 1.16, 95% CI: 1.12–1.20) and NCD education (OR = 1.24, 95% CI: 1.20–1.29) but were somewhat less likely to choose CHCs for care (OR = 0.93; 95% CI: 0.88–0.99). Willingness to Integrate: Migrants with a higher willingness to integrate were more likely to receive health education for both IDs (OR = 1.27; 95% CI: 1.21–1.34) and NCDs (OR = 1.28: 95% CI: 1.22–1.35) but tended to avoid CHCs (OR = 0.89; 95% CI: 0.83–0.96). Perceived Hygiene Habit Similarity: Migrants who perceived their hygiene habits as similar to locals were more likely to receive health education on both IDs (OR = 1.14; 95% CI: 1.07–1.21) and NCDs (OR = 1.13, 95% CI: 1.06–1.20) and were also more likely to use CHCs (OR = 1.14; 95% CI: 1.02–1.27).
Ding et al. (87)	3,412 pregnant women migrants (2014 CMDS)	Cross-sectional study	Social integration was measured through economic integration (employment status, monthly household income, housing in the current residence); acculturation (duration of stay in current residence), and identification (willingness to stay)	Hospital childbirth at hometown	Family members in current residence, Hukou, establishment of health records, migration reason, social medical insurance status, education level	Economic Integration: Migrant women with their own housing in the destination area are less likely to return to their hometowns for childbirth (OR = 0.351; 95% CI: 0.207–0.595). Cultural Adaptation: Those residing for 5 years or more are less likely to return home for childbirth (OR = 0.449; 95% CI: 0.322–0.626). Self-Identity: Migrant pregnant women who are willing to live in the destination area long-term are less likely to return home for childbirth (OR = 0.731; 95% CI: 0.537–0.995).
Peng et al. (88)	4,018 migrants who were hospitalized in the past year (2014 NIMDMS)	Cross-sectional study	Social integration was measured through economic integration (employment status, monthly household income, housing in the current residence) and self- identification (willingness to stay)	Medical return	Age, gender, ethnic group, education level, marital status, and household size in current residence; economic development of current residence, migration type, reason for migration, and duration of staying in current residence	Rural-to-urban migrants enrolled in the NRCMS of hometown preferred inpatient service in hometown (OR of hometown vs. residence=2.44, 95%CI 1.80–3.30); Migrants employed also preferred hometown (OR = 1.29, 95%CI: 1.05–1.60). Permanent settlement intention was positively associated with being hospitalized in the current areas of residence (OR =0.66, 95%CI: 0.48–0.90)
Zhao et al. (89)	627 MEFC in Jinan city, Shandong Province	Cross-sectional study	Social integration was measured through economic integration (local medical insurance status), acculturation (duration of stay in current residence), and identification (willingness to stay for a long time).	Medical return	Age groups, gender, ethnic group, education level, marital status, household monthly income, Hukou, migration reason, migration range, applied for temporary resident permit, number of accompany-migrated	MEFC who have not joined the local medical insurance are more likely to return to their hometown for medical services (OR = 3.561; 95% CI: 1.577–8.039); MEFC who stated their low willingness to stay in the inflow area have higher possibility on medical returns (OR = 2.600, 95% CI: 1.620–4.174).
**SRH**						
Lin et al. 2016 (90)	1,999 migrants and 1,997 local residents in Zhongshan city	Cross-sectional study	Social integration measured through four dimensions: economic status (employment, labor contract, household income, daily working time, subjective social status and level of respect), social communication (organizational members, participation in social activities), acculturation (attitude toward social norms, language used), and self-identity (willingness to interact with local residents; self-identification as a local resident)	SRH	Gender, age, education and marital status	Economic integration aspects: income and relative socioeconomic status (β = 0.259, *p <* 0.001) and degree of respect received from others (β = 0.339, *p <* 0.001) was significantly positively related to SRH; Social communication: social activity participation (*β =* 0.288, *p <* 0.05) Self-identity: stronger integration will (*β =* 0.083, *p <* 0.001) and identifying themselves more as non-locals (*β =* −0.550, *p <* 0.05) was associated with SRH
Wei et al. (91)	656 MEFC ≥60 years old in Jinan city, Shandong Province	Cross-sectional study	Social support, assessed by Social Support Rating Scale	SRH	Sex, age, BMI, monthly income, education, employment, migration years, migration type, presence of an elevator, living condition, morbidities.	MEFC who lived with family were less likely to report decreased levels of health (OR = 0.033, *p*= 0.010). However, support from the external social network is not significantly associated with SRH (*p* > 0.05).
Lu et al. (92)	117,466 migrants (2017 CMDS)	Cross-sectional study	Social integration measured by 8 questions: (i) “I like this city”; (ii) “I noticed the changes in this city”; (iii) “I am willing to integrate into this city”; (iv) “The local residents are willing to accept me”; (v) “I have not been discriminated against by local citizens”; (vi) “Following the customs of my hometown is not important to me”; (vii) “My personal hygiene habits are not different from those of local citizens”; and (viii) “I am already a local resident.”	SRH	Gender, age, education, marriage status, length of stay in the city, household migration status, the logarithm of the monthly wage, weekly work hours, medical insurance status, and social ties	The positive and significant effect of social integration on the SRH of rural-to-urban migrants remains consistent—-one standard deviation increase in social integration corresponds to a 0.072 standard deviation improvement in SRH. SRH of women (SD = 0.096, *p <* 0.01) and the younger-generation (SD=1.198, *p <* 0.01) migrants benefitted significantly from social integration.
Lin et al. (72)	15,999 migrants from 8 cities (2014 NIMDMS)	Cross-sectional study	Social integration was measured through socioeconomic status, social interaction, culture adoption, and integration willingness	SRH	Age, gender, education, Hukou, marital status, years of residence, social insurance	Acculturation and integration will is significantly positively associated with SRH (*β =* 0.324, *p <* 0.05); Gini × Socioeconomic status significantly negatively associated with SRH (*β =*−0.683, *p <* 0.001)
**NCD & SRH**						
Zhu et al. (93)	830 migrants and 216 local residents in Shenzhen city	Cross-sectional study	Structural social capital: participation in organizations and social contacts	Hypertension control; SRH	Age, gender, education, occupation, year of hypertension and family history of hypertension	Migrant hypertensive patients have lower structural social capital in terms of social contacts (10.87 vs. 10.41; *β =* −0.457, 95% CI: −0.866 to −0.048) and poorer blood pressure control (56.4 vs. 43.6%; OR = 0.557, 95% CI: 0.364–0.852) when compared to the local individuals; Lower structural social capital is associated with worse SRH (OR = 0.861, 95% CI: 0.788–0.941). However, no significant difference in the association between structural social capital and SRH was observed between the migrants and natives.

NIMPDMS, National Internal Migrant Population Dynamic Monitoring Survey; OR, odds ratio; CI, confidence interval; CMDS, China Migrants Dynamic Survey; MEFC, migrant older adult following children; SD, standard deviation.

### 3.3 Quality appraisal results of included studies

The quality score of the included studies ranged from 12 to 16 using the *JBI Critical Appraisal Checklist* (Table 3). Seven studies were rated as having moderate quality and the other 9 were rated as high-quality studies. The average quality rating score was 14.1. These results indicate a generally favorable quality of the evidence, suggesting that the subsequent results of our systematic review are reliable and robust.

**Table 3:** Quality assessment of the included studies (*N* = 16).

**References**	**Criteria for inclusion**	**Description of study subjects & setting**	**Measurement of exposure**	**Measurement of condition**	**Identification of confounders**	**Method to control confounders**	**Measurement of outcomes**	**Use of statistical analysis**	**Overall score**	**Quality rating**
Hou et al. (79)	2	2	2	2	2	2	1	2	16	High
Hu et al. (80)	2	2	2	2	1	1	2	2	15	High
Jiang (81)	1	1	2	2	2	1	2	2	14	High
Jing et al. (82)	2	1	2	2	1	1	2	1	12	Moderate
Liang et al. (83)	2	2	2	2	2	2	2	2	16	High
Lin, Y et al. (84)	2	2	1	2	1	1	1	2	12	Moderate
Wang et al. (85)	2	2	2	2	1	1	2	2	14	High
Wang et al. (86)	1	2	2	2	2	1	2	1	13	Moderate
Ding et al. (87)	1	2	1	2	1	2	2	2	13	Moderate
Peng et al. (88)	2	2	2	2	2	2	2	2	16	High
Zhao et al. (89)	2	2	2	2	1	2	2	2	15	High
Lin et al. (90)	2	2	2	2	1	2	2	2	16	High
Wei et al. (91)	2	2	1	2	1	1	2	2	13	Moderate
Lu et al. (92)	1	1	2	2	2	1	2	2	13	Moderate
Lin et al. (72)	1	1	2	2	1	1	2	2	12	Moderate
Zhu et al. (93)	2	2	2	2	2	1	2	2	15	High

Each criteria were assigned a score of 2 (fully met the criteria), 1 (partially met the criteria), or 0 (did not met the criteria or not applicable), respectively, and the summed total score was between 0 and 16. Then, each study was categorized as high quality (14–16 points), moderate quality (9–13 points) or low quality (0–8 points).

In this assessment of 16 included studies, a notable trend emerged regarding the performance across different domains. The *Criteria for Inclusion* and *Description of Study Subjects & Setting* were consistently strong, with more than 10 studies achieving high ratings in these areas, demonstrating a rigorous approach to participant selection and thorough demographic reporting. The *Measurement of Condition* consistently outperformed, receiving a score of two across all studies, indicating satisfying quality on how conditions were measured and reported. The *Identification and Control of Confounders* showcased variability; while seven studies identified confounders effectively, only six implemented methods to control these confounders adequately. This inconsistency highlights an area for improvement in study designs to enhance the reliability of the findings. Additionally, the *Measurement of Outcomes* showed mixed evaluations, with two studies scoring lower, suggesting that outcome measurement could benefit from standardization or more rigorous methodologies. Finally, the application of *Statistical Analysis* was generally well-executed, with 14 studies performing statistical analyses to support their findings, further contributing to the overall validity of the results. In summary, while many studies demonstrate high quality, there remains room for improvement, particularly in the areas of confounder management.

### 3.4 Measures of social integration

Social integration was measured using multi-dimensional frameworks and a high level of heterogeneity was observed between the studies regarding the measurement of social integration, although the detailed item might vary. These dimensions encompassed (i) economic integration, measured by local employment, monthly income, local social and medical insurance status and local housing status; (ii) acculturation, measured by length of time lived in the inflow region and preferred customs; (iii) social involvement, including participation in local activities and memberships, and number of local friends; and (iv) self-identification, measured by integration and settlement willingness. Four studies have examined social integration both subjectively and objectively.

### 3.5 Association between social integration and healthcare service utilization

Healthcare service utilization was extensively examined in 11 studies, focusing on the relationship between social integration and various dimensions of healthcare use, including health record establishment, health education, preventive services, community health center utilization, and medical return behavior. Social integration was consistently found to be a significant factor influencing healthcare service use among rural-to-urban migrants. The key components of integration, such as economic stability, social networks, cultural adaptation, and self-identity were identified as strong predictors of better access and utilization of healthcare services.

#### 3.5.1 Health record establishment

Several studies highlighted the positive association between social integration and personal health record establishment. Those who proactively joined in social organizations and activities are more likely to have their health record established in the host region (Hou et al.: OR_social organization =_1.467, 95% CI: 1.201–1.793; OR_social activities_ = 1.620, 95% CI: 1.329–1.976; Jiang Junfeng: OR_community work_ = 1.241; OR_donation/volunteering_ = 1.275; OR_political party_ = 1.058; *p* < 0.001; and Liang et al., OR_social organization =_1.618, 95% CI: 1.576–1.660) (79, 81, 83). Having local medical insurance was strongly associated with the establishment of health records, that older adult migrants with local medical insurance were twice as likely to have health records (OR = 2.03, 95% CI: 1.60–2.57) compared to those without insurance (82). For married female migrants, their greater ability to utilize surrounding social support was linked to a higher likelihood of health record establishment (OR = 1.24; 95% CI: 1.06–1.44) (80). These associations suggest that enhancing social integration is crucial for improving health record establishment among migrant populations.

#### 3.5.2 Health education

A comprehensive set of social integration indicators were associated with the receipt of health education. Study by Liang, J. et al. found that migrants who were employed (OR = 1.121, 95% CI: 1.081–1.163), participated in social organizations (OR = 1.700, 95% CI: 1.658–1.744), engaged in civil activities (OR = 1.535, 95% CI: 1.497–1.574), had local medical insurance (OR = 1.064, 95% CI: 1.033–1.095), maintained local friendships (OR = 1.146, 95% CI: 1.114–1.179), or expressed a willingness to settle in their current location (OR = 1.180, 95% CI: 1.141–1.220) were more likely to receive health education on infectious diseases (IDs); similarly, factors such as owning a local house (OR = 1.048, 95% CI: 1.016–1.081), participating in social organizations (OR = 1.807, 95% CI: 1.763–1.851), civil engagement (OR = 1.605, 95% CI: 1.566–1.644), local medical insurance (OR = 1.049, 95% CI: 1.020–1.079), local friendships (OR = 1.150, 95% CI: 1.119–1.182), and a willingness to settle (OR = 1.172, 95% CI: 1.135–1.211) were significantly associated with receiving health education on NCDs (83). Wang et al. did not only yielded similar associations in their study, but also discovered migrants who experienced the least influence from hometown customs were significantly more likely to receive health education on both IDs (OR = 1.16; 95% CI: 1.12–1.20) and NCDs (OR = 1.24; 95% CI: 1.20–1.29). Similarly, migrants who agreed their hygiene habits similar as those of local residents were more likely to receive health education on both IDs (OR = 1.25; 95% CI: 1.17–1.33) and NCDs (OR = 1.18; 95% CI: 1.11–1.26) (86). The results show that being socially connected is crucial in how migrant populations access health education, with community support and local networks playing a key role in improving health literacy.

#### 3.5.3 Preventive health services

Preventive health services utilization, including vaccinations, health check-ups and cancer screenings, was strongly correlated with social integration. Migrants with robust social capital were more likely to engage in preventive measures. For instance, actively reaching out to local social capitals were linked to higher odds of contraceptive use consultations (OR = 1.21; 95% CI: 1.04–1.42) and cancer screenings (OR = 1.29; 95% CI: 1.10–1.51) among migrant women who had a spouse (80). Having local friends significantly increased the likelihood of receiving free health check-up at the community health centers by 2.859–4.607 times (*p* < 0.001), which service is a national program designed for the older adult population than those without local peers (84). In addition, improved social integration status was positively associated with the participation in the influenza vaccination program (OR = 1.142; 95% CI: 1.04–1.22) (85). The findings highlight the essential role of social integration in facilitating access to preventive health services.

#### 3.5.4 Community health center utilization

Community health centers (CHCs) served as vital and first-point-of-access for primary care. Study conducted by Wang et al. found that migrants engaged the most in civil activities (OR = 2.61; 95% CI: 1.49–4.58) and perceived least differences in hygiene habits compared to local residents (OR = 1.14; 95% CI: 1.02–1.27) were more likely to utilize CHC services (86). However, the other aspects of acculturation as well as self-identity were found to hinder CHD utilization, as those who with strong local identification (OR = 0.66; 95% CI: 0.62–0.70), preferred the host region over their hometown (OR = 0.96; 95% CI: 0.91–1.00), and influenced more by inflow region customs (OR = 0.93; 95% CI: 0.88–0.99) were significantly less likely to visit CHCs as their primary healthcare institution when they got sick (86). The outcomes indicated a complex interaction between social integration and acculturation, suggesting that both community engagement and identity perceptions significantly influence the utilization of CHCs among migrant populations.

#### 3.5.5 Medical return behavior

Three studies examined the phenomenon of medical return in specific migrant subgroups, where they traveled back to their hometowns for healthcare, rather than seeking healthcare in the inflow region. Economic integration was a significant influence factor, that having an own home is significantly associated with decreased likelihood of medical return among the pregnant women migrants (OR = 0.351; 95% CI: 0.207–0.595), while haven't joined the medical insurance scheme at the host region was significantly related to the medical return of the MEFCs (OR = 2.44, 95% CI: 1.80–3.30) and who needed to be hospitalized (OR = 3.561; 95% CI: 1.577–8.039) (87–89). Interestingly, being employed in the inflow place is associated with higher chances in receiving hospitalization service at hometown (OR = 1.29, 95% CI: 1.05– 1.60) (88). Willingness to permanently settle in the inflow area is linked to reduced medical returns for childbirth in pregnant women (OR = 0.731; 95% CI: 0.537–0.995) and migrant patients who needed to be hospitalized (OR = 0.66, 95% CI: 0.48–0.90) (87, 88), while reduced settlement intention significantly increase possibility to return to the older adult's original place for healthcare service (OR = 2.600, 95% CI: 1.620–4.174) (89). These findings emphasize that both personal circumstances and community ties significantly influence decisions regarding medical care of the migrants.

### 3.6 Association between social integration and SRH

In our systematic review, five studies investigated the impact of social integration on SRH among the internal migrant populations. One study measured social integration through an 8-Likert scale and the result found one standard deviation increase in the social integration score was associated with a 0.072 standard deviation improvement in SRH (*p* < 0.01), with women (SD = 0.096, *p* < 0.01) and the younger-generation (SD = 1.198, *p* < 0.01) as the most significantly benefitted migrant subgroups from increased social integration (92). The study by Lin et al. recruited 1,999 migrants and indicated that economic integration, especially wage and the social standing of the occupation (β = 0.259, *p* < 0.001) and the relative respect level in their social network (β = 0.339, *p* < 0.001) were significantly associated with SRH of the migrants; active participation in social activities (β = 0.288, *p* < 0.05) and strong integration will (β = 0.083, *p* < 0.001) are related to increased SRH, while those who identify themselves more as outsiders are linked to decreased SRH (β = −0.550, *p* < 0.05) (90). Another study examined the mixed effect of socioeconomic inequality, measured by Gini coefficient, and social integration status on the migrant's health and made comparison between eight cities in China, that the outcome showed migrants in cities with lower income inequality reported significantly higher SRH compared to those in cities with greater inequality (β = −0.683, *p* < 0.001) (72). Older adult migrants who followed their children and live with the family in the inflow city reported better SRH (OR = 0.033, *p* = 0.010), whereas social support externally from friends, relatives, colleagues or neighbors did not show a significant impact (*p* > 0.05) (91). As for migrants and local residents who were diagnosed with hypertension, their structural social capital did not have an impact on their SRH (93). Overall, the results illustrate the critical role of social integration in enhancing self-reported health among internal migrants.

### 3.7 Association between social integration and NCDs

In addition to healthcare service utilization and SRH, our review also explored the relationship between social integration and NCDs. The systematic review only identified one study that examined the relationship between social integration and NCDs and comparisons were made among migrants and the native residents in Shenzhen city. Migrants demonstrated significantly lower structural social capital, demonstrated by a reduced number of social contacts (mean score: 10.41 vs. 10.87, β = −0.457; 95% CI: −0.866 to −0.048), while the hypertension among migrant is 45% less likely to be controlled well when compared to the local people (OR = 0.557; 95% CI: 0.364–0.852) (93). These results suggest that enhancing social integration may be crucial for improving NCD management in migrant populations.

## 4 Discussion

This review provides a comprehensive overview of the social integration and health outcomes of the internal migrant population in China, where social integration indicators in the included studies were measured extensively using multiple dimensions, including economic integration, interpersonal relationships, social support, self-identity, and cultural adaptation, allowing a wide range of the associations between social integration and various health outcomes to be explored. At the same time, these studies not only focus on the general migrant group that is prone to health inequality, but also pay further attention to the more vulnerable older adult migrants, especially those who followed their migrant children into the inflow region; and migrant women, including those who were pregnant or married. Notably, the primary themes discussed in our systematic review include health service utilization, SRH, and NCDs as critical health outcomes for the internal migrant population in China.

### 4.1 The transformative role of social integration in healthcare service utilization

In this systematic review, the majority of the included articles investigated the relationship between migrants' social integration and health service utilization, including health record establishment, health education reception, preventative disease management and utilization of primary health services in the inflow areas. These health care offerings are core parts of the “basic public health service program” and “major health service program” implemented by China National Health Commission in promoting the national strategy of universal health coverage (94). The common finding across these studies is that the migrants who actively participate in in various social activities, have a local social network, consider themselves to be more like a part of the host community in terms of identity and habits, have affection for the place of destination with long-term settlement tendencies, and whom own local housing and medical insurance are significantly more likely to establish health records, receive health education, and accept health screening and vaccination services. Engagement with social networks and participation in social activities provide migrants with greater access to information about healthcare services. A Canadian study pointed out that immigrants' medical behavior is affected by the community and population they are surrounded by (95). Study by Litwin et al. indicated that those who have more comprehensive social network used healthcare service more frequently compare to those who had a family-oriented network (96).

A strong sense of belonging to the place of destination makes migrants more trustful of local healthcare resources, while their identity as “one-of-the-locals” also makes them more confident and acquainted when accessing these services, without feeling discriminated as “outsiders” (97, 98). Moreover, better economic integration, exemplified by home ownership and medical insurance, contributes to greater stability for the internal migrants, providing a safety net when they are faced with medical issues and helping to prevent a return to poverty due to illness. Study by Zhang et al. demonstrated that after the implementation of NRCMS, the recent decade has seen a leap in their healthcare service utilization by the rural population in China, and a significant reduction of their out-of-pocket money and greatly improved the affordability of healthcare services (99). Under China's tiered healthcare reform, CHCs serve as the gatekeepers of the primary health care system, where residents can obtain a variety of basic diagnosis, treatment, public health, and preventive services, and can receive higher medical insurance reimbursement compared with the higher tier health institutions (100). However, one of the included articles found that various indicators of social integration had mixed effects on migrants' use of CHCs (83). This may be due to the presence of numerous medical institutions at all tiers in the areas to which migrants flow, as the current reform does not restrict the level of residents' initial healthcare facilities. Consequently, while some prefer nearby CHCs, others will choose higher-tier medical institutions for healthcare services.

Our review includes relevant evidence regarding the medical return behaviors of different groups of migrants with medical needs, which also reflects the phenomenon of medical non-utilization in the destination region. In the salmon bias hypothesis, immigrants will leave the country of immigration and return to their place of origin after health disadvantages occurred (101). Our study is consistent with the hypothesis and has further explored the role of social integration in this event. Economic integration, such as participation in the medical insurance scheme at the inflow area is one of the key factors affecting the choice of medical return by migrants, which further explains the importance of universal health coverage, simplifying the reimbursement procedures and enhancing the reimbursement rate for medical insurance at the inflow area for the migrant populations (102). Instability of residence in the place of inflow, such as not owning a home, shorter residence time and lower willingness to stay long-term are also significantly related to medical return. One exception is that migrants employed in the destination area show a higher tendency to return for medical treatment, which may be due to the fact that many jobs held by migrants has a temporary or seasonal pattern. Furthermore, in traditional Chinese culture, family plays a crucial role in patients' medical decisions and caregiving, making them more inclined to return to an environment where their families reside (103).

It is noteworthy that among internal migrants experiencing medical return, older adult and female migrants are particularly represented, as they are often considered vulnerable subgroups within the internal migrant community. For aged internal migrants, a cohesive and family-centered network, their main source of social support in the host community, can reduce social isolation, a major risk factor for both physical and mental health decline among the older adults. Study has shown that older adult internal migrants in China often face significant challenges in cultural adaptation to the host region. However, support from their families and hometown can help create a familiar environment to some extent, facilitating their cultural adaptation (104). This study also found that female migrants are more likely to engage in medical return for childbirth when they lack economic security and a sense of belonging in the host region, even when better healthcare services are available there. The absence of financial stability and social support influences their access to maternal healthcare and contributes to increased psychological stress and anxiety during pregnancy and childbirth. These findings illustrate how social support, economic security, and cultural adaptation influence the medical return behaviors of older adult and female migrants, reflecting their unique challenges in accessing and utilizing healthcare.

### 4.2 The pivotal influence of social integration on SRH

SRH is a reliable indicator for predicting morbidity and mortality in the population (105, 106). Our study found that social integration was significantly and positively associated with migrants' SRH in four out of five studies included. Similar to healthcare service utilization, in-depth economic integration, cultural adaptation and self-identification as natives were again found to be related to improved SRH. This finding aligns with results from international studies, which demonstrate that social integration plays a crucial and positive role in both physical and mental health, such as improving the prognosis of major diseases, encouraging regular physical activity and balanced dietary habits, and reducing unhealthy lifestyles (107, 108). Furthermore, social integration significantly alleviates loneliness and decreases the prevalence of psychological issues such as depression, all of which contribute positively to SRH (109). Therefore, social integration is an important influencing factor in improving the health of migrants. Another study focusing on MEFC did not find an association between external social integration and their SRH; instead, only family relationships were significantly associated with their SRH (91). Intergenerational support is a cornerstone of traditional Chinese family culture. Migrant older adult individuals often relocate to join their migrant children in urban areas, where they take on caregiving responsibilities, such as managing household chores and looking after grandchildren (110). However, their own wellbeing is often overlooked as many in this group report a lack of belonging, feelings of loneliness, and social isolation in their new environment (111). While the included studies did not establish a direct link between insufficient social integration and reduced SRH among migrant older adult individuals, these conditions may, over time, lead to challenges at the individual, family, and societal levels. This finding emphasizes the need to address the unique health needs of this specific group in destination areas.

### 4.3 The impact of social integration on NCD management

Only one study regarding the relationship between social integration and NCD was included in our review, which revealed that migrants had significantly less accountable interpersonal relationships and worse hypertension control, compared to the local residents (93). This may be attributed to the limited social interactions migrants experience upon settling in a new environment (112). Combined with their relatively disadvantaged socioeconomic status, this often restricts their access to essential medical resources for effective blood pressure management. Although China's internal migrants have a relatively lower prevalence of NCDs, their poor awareness, prevention, and control of NCDs may lead to even more serious consequences (113–115). This issue requires attention, as migrants represent a tremendous population and are also facing the aging issue, similar to the general population in China.

### 4.4 Pathways of social integration in shaping migrant health outcomes

The conceptual framework on the relationship between social networks and health by Berkman et al. proposed that social networks serve as a critical determinant of health outcomes, with psychological support being a key mediating factor (41). Psychological support enhances migrants' ability to cope with stress, reduces depressive symptoms, and strengthens resilience and overall wellbeing (116). The formation of strong emotional bonds within the host community can further amplify these benefits, even surpassing the impact of tangible support (117). In contrast, lacking of proper psychological support may influence individuals' physical health, such as dysregulate neuroendocrine function, weaken immune defenses and impair cardiovascular function, thereby increasing susceptibility to illness, elevating morbidity risks, and potentially reducing life expectancy (41, 118). Another key pathway through which social integration influences migrant health is economic improvement, which facilitates healthcare access, alleviates financial stress, and strengthens health literacy (63, 119). Economic stability, such as securing stable employment and obtaining local health insurance in the host region, reduces financial barriers to medical services, supports better housing and nutrition and increases access to preventive care and timely treatment (72, 120, 121). Moreover, income improvement plays a crucial role in reducing health disparities between migrants and local residents by narrowing gaps in healthcare affordability, demonstrating the broader health benefits of social integration (72). Another crucial mechanism linking social integration to migrant health is accessibility to information (51, 122). As migrants become more embedded in local networks, they gain critical knowledge about healthcare services, public health programs, and disease prevention (123). This increased awareness translates into greater healthcare utilization, earlier disease detection, and healthier lifestyle choices (124). Moreover, improved access to reliable health information equips migrants to navigate healthcare systems with confidence, mitigating the risks posed by misinformation and fragmented care. By closing the informational gap between migrants and local residents, social integration not only enhances health equity but also magnifies the transformative effects of economic stability, collectively driving a more inclusive healthcare landscape. Together, these interconnected pathways demonstrate that social integration is a fundamental determinant of migrant health, working through psychological, economic, and informational channels to reduce health disparities and improve health outcomes.

### 4.5 Analysis of strengths and limitations

This systematic review represents the first comprehensive synthesis of evidence regarding the relationship between social integration and health outcomes among internal migrants in China. By incorporating 16 studies, most of which were national-level databases, and employing multidimensional frameworks to assess social integration, this review explores a wide range of health outcomes, including healthcare service utilization, self-reported health, and noncommunicable diseases. A notable strength is the inclusion of vulnerable subpopulations, such as older adult migrants and female migrants which highlights the varied impacts of social integration on different demographic groups. Additionally, the use of robust quality assessment tools ensures that the studies included meet high methodological standards. However, this review also has limitations. All the included studies applied a cross-sectional design, which restricts causal inferences about the relationship between social integration and health outcomes. Future research should employ longitudinal or intervention-based designs to better capture the dynamic relationships between social integration and health over time. Moreover, the heterogeneity in how social integration was measured across studies posed challenges for synthesis and comparison, as different dimensions were not uniformly assessed, emphasizing the necessity for standardized assessment frameworks that allow for more consistent comparisons. The findings are context-specific and may not be generalized to other countries or regions with different migration patterns and health systems. Expanding research to an international context would provide a broader perspective on how social integration influences migrant health across diverse settings. Additionally, while this review focuses on social integration, other potential confounders, such as occupational status, cognitive health, and family structures, may also influence health outcomes of the internal migrants. Future studies should explore how these factors interact with social integration to impact migrant health, particularly in the context of China, where research on these interconnections remains scarce. Furthermore, this review relies on traditional systematic review methods without incorporating newer approaches, such as mixed-methods that integrate quantitative and qualitative insights (125); or social network analysis, which reveal the impact of social connections on health (126). Research should explore these advanced methodologies to deepen the understanding of social integration and migrant health in the future.

### 4.6 Research significance and future prospects

Improving social integration and health outcomes for internal migrants requires coordinated efforts at national, urban, and community levels. Reducing health inequalities, enhancing healthcare accessibility, and strengthening disease management shall be prioritized to improve overall health among migrant populations. At the national level, reforms to the Hukou system are essential to enhance access to healthcare services and social welfares. Expanding universal health coverage insurance and creating unified reimbursement systems can ensure migrants receive equitable care across regions, particularly in managing chronic diseases that require long-term treatment and continuity of care. National policies should also promote migrant-friendly healthcare infrastructure to reduce disparities in medical service accessibility. Additionally, national campaigns should also promote inclusivity and highlight the importance of migrant health for societal wellbeing.

At the urban level, cities should integrate migrant needs into urban planning by increasing affordable housing, expanding culturally tailored health education, and establishing health facilities in migrant-dense areas. Improving access to preventive healthcare services, particularly in early screening and chronic disease management, is essential to reducing long-term health disparities. Strengthening urban healthcare networks to serve migrants effectively can help mitigate barriers to primary care and specialist treatment. Policies that encourage migrant participation in civic activities can foster a sense of belonging and strengthen their social networks.

At the community level, grassroots initiatives can bridge gaps between internal migrants and locals by organizing social events, volunteering programs, and peer support groups. Community health programs should be strengthened to ensure migrants have access to essential healthcare services, including chronic disease prevention, mental health support, and maternal care. Mobile health units, digital health tools, and telemedicine services could further improve accessibility for migrants facing geographic or financial barriers. Establishing resource centers to provide health education, legal aid, and vocational training can further enhance integration while addressing migrants' specific needs.

Furthermore, future research should focus on the specific health challenges faced by vulnerable internal migrant subgroups, particularly older adult and female migrants, which remain underexplored in the current literature. Studies should investigate the role of social support and cultural adaptation in influencing health outcomes for older adult migrants, especially examining how family and community networks influence their health. Additionally, the reproductive and mental health of female migrants warrants more attention, with a focus on how migration impacts access to maternal care, reproductive health decisions, and psychological health. Improving healthcare accessibility for older adult and female migrants through targeted health policies and community-based interventions will be essential in reducing inequalities and ensuring comprehensive care.

Targeted social integration programs should be implemented to ensure that these vulnerable migrant subgroups can access quality healthcare in the host region, thereby improving their overall health and reducing disparities in health service utilization. This multi-tiered approach requires diminishing health disparities among migrants and extends beyond the healthcare system and call for a comprehensive societal response. Coordinated efforts across national, urban, and community levels can foster an environment that enhances social integration and reduces health inequities, ultimately supporting the wellbeing and inclusion of internal migrants in their host communities.

## 5 Conclusion

This systematic review demonstrates that social integration significantly influences the health outcomes of internal migrants in China. Economic stability, cultural adaptation, and social networks emerge as key determinants of healthcare service utilization, self-reported health, and chronic disease management. Policymakers should prioritize reforms to improve economic inclusion, enhance medical insurance coverage, and promote community-based support to improve social integration and reduce health disparities for the internal migrant populations in China, and ultimately contribute to a more equitable and healthier society for all.

## Data Availability

The original contributions presented in the study are included in the article/supplementary material, further inquiries can be directed to the corresponding author.
